# First-in-human nuclease-free homologous recombination-dependent gene editing in pediatric patients with methylmalonic acidemia: results of a phase 1/2 study

**DOI:** 10.1038/s41434-026-00609-1

**Published:** 2026-03-30

**Authors:** Jirair K. Bedoyan, Thomas Morgan, Angela Sun, Hong Li, Daniel Gruskin, Marie Payton, Frederic Chereau, Eugene Scott Swenson, Qun Lin, Mark A. Kay, Jerry Vockley

**Affiliations:** 1 UPMC Children’s Hospital of Pittsburgh, Pittsburgh, PA USA; 2 University of Pittsburgh School of Medicine, Pittsburgh, PA USA; 3 Vanderbilt University School of Medicine, Nashville, TN USA; 4 Seattle Children’s Hospital, Seattle, WA USA; 5 Emory University, Atlanta, GA USA; 6 LogicBio Therapeutics, Lexington, MA USA; 7 Alexion, AstraZeneca Rare Disease, Boston, MA USA; 8 Stanford University, Stanford, CA USA

**Keywords:** Metabolic disorders, Gene therapy

## Abstract

Gene-based editing can potentially correct the genetic defect in methylmalonic acidemia (MMA). SUNRISE, a first-in-human phase 1/2 open-label study, evaluated the safety/tolerability (primary endpoints) of liver-targeted hLB-001 in four pediatric participants (ages 20-114 months) with mitochondrial methylmalonyl-CoA mutase (MMUT)–deficient MMA. We designed a single-infusion adeno-associated viral capsid (hLB-001) to nondisruptively integrate functional *MMUT* at the 3′ end of the albumin (*ALB*) locus to produce both albumin and MMUT. All four participants experienced at least one treatment-emergent adverse event. Three participants had treatment-emergent serious adverse events of cytokine release syndrome (one participant) and thrombotic microangiopathy (two participants); all resolved during the trial. Biologic activity, clinical efficacy, and 1-year survival were secondary endpoints. MMUT expression (measured by 2A-tagged ALB biomarker expression) increased in two participants over two years, confirming homology-based integration and positive selection of transgenic cells. However, serum methylmalonic acid (sMMA), serum FGF21, serum methylcitric acid (sMCA), and propionate oxidation remained abnormal in all four participants. All participants were alive at 1 year and at database lock. SUNRISE was terminated due to lack of efficacy. These results provide proof-of-concept for use of liver-targeted gene editing without nucleases for MMA and other genetic metabolic disorders. ClinicalTrials.gov identifier: NCT04581785

**Target journal**: *Gene Therapy (Springer Nature)*

## Introduction

Isolated methylmalonic acidemia (MMA) is an inborn error of metabolism caused by variants in the mitochondrial methylmalonyl-CoA mutase (*MMUT*) gene, resulting in an inability to metabolize certain amino acids and fats [1, 2]. It is inherited in an autosomal recessive pattern with an incidence of approximately 1 in 100,000 and 1 in 160,000 live births in the European Union and United States, respectively [2–4].

MMA affects multiple organs (e.g., central nervous, renal, cardiac, endocrine, gastrointestinal, hepatic, hematologic systems) and various functions (e.g., cognition, movement, vision, hearing, feeding, bone health) [2, 5, 6]. Individuals with severe MMA are at increased long-term risk for neurologic injury (e.g., metabolic stroke, seizure), poor feeding, and failure to thrive [6]. Disease progression can also lead to developmental delay, impaired cognition, and renal failure, with devastating impacts on an individual’s quality of life and survival [5, 6].

Diagnostic biomarkers include elevated methylmalonic acid in the blood and urine, presence of 3-hydroxypropionate and 2-methylcitrate in urine, and elevated propionylcarnitine in blood [6]. Other features associated with MMA include elevated fibroblast growth factor 21 (FGF21) in plasma, hyperammonemia, ketosis, and hyperlactatemia as well as anemia, neutropenia, and/or thrombocytopenia [2, 6]. Infants with severe MMA are diagnosed in the first days to weeks of life based on either newborn screening results or presentation with clinical signs, including vomiting, lethargy, respiratory distress, hypotonia, hypothermia, and progressive encephalopathy [1, 2, 6]. MMA can also be diagnosed by prenatal genetic testing in at-risk pregnancies [6].

Currently, MMA has no curative therapies. Individuals with MMUT-deficient MMA are treated with a lifetime protein-restricted diet, in addition to interventions to mitigate disease-associated factors contributing to metabolic brain injury, renal failure, and liver toxicity [2, 6]. Liver transplantation and combination liver/kidney transplantation are established interventions in severe MMA that are increasingly performed at an early age to suppress acidotic and hyperammonemic episodes, stabilize neurocognitive function, and improve long-term survival [1, 2, 6, 7]. However, donor organ availability is limited, and transplantation has significant acute and long-term risks associated with both the surgical procedure itself and lifelong immunosuppression [1, 7, 8]. Gene editing therapy has the potential to correct the underlying genetic defect in MMA, restoring sustained expression of hepatic MMUT enzymatic activity and transforming lives for individuals with MMA and their caregivers [9, 10].

**Fig. 1:**
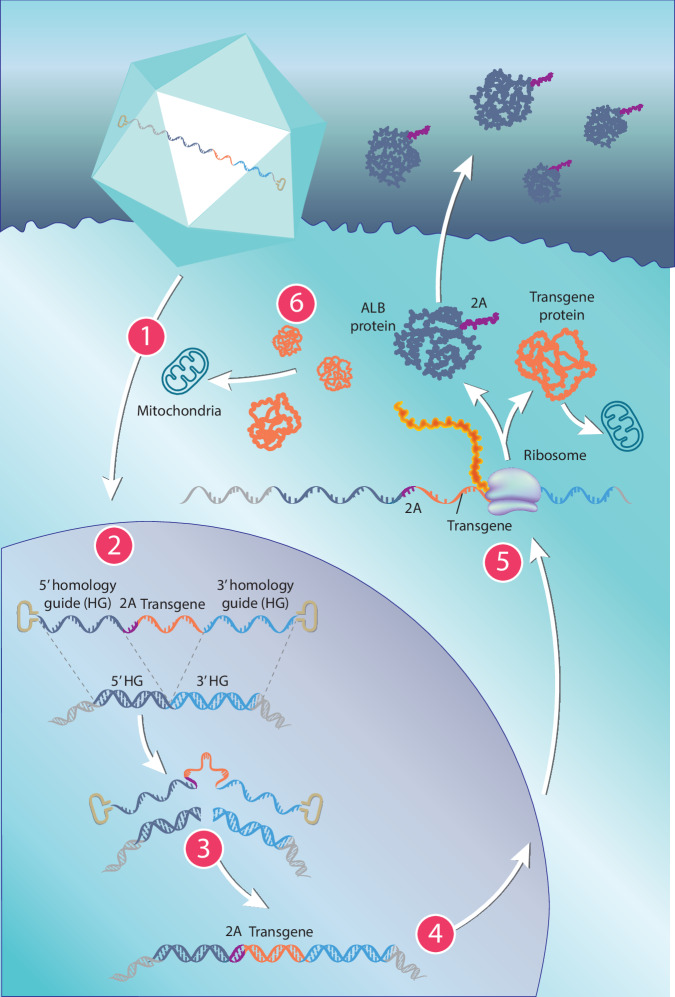
hLB-001 mechanism of action. **1** hLB-001 utilizes the AAV-LK03 capsid to deliver a functional copy of the human *MMUT* coding sequence for nondisruptive integration into the 3′ end of the *ALB* locus of hepatocytes. **2** The AAV-LK03 capsid directs delivery of the genetic sequence to the nucleus of hepatocytes. **3** The human *MMUT* gene is integrated into the *ALB* locus via homologous recombination. **4** Following transcription, **5** the P2A sequence induces ribosome skipping, leading to production of ALB and MMUT as two separate proteins. **6** The ALB-2A protein is released into circulation and serves as a biomarker of *MMUT* integration at the targeted *ALB* locus, and transgenic MMUT is directed to the mitochondria. AAV adeno-associated virus, ALB albumin, MMUT methylmalonyl-CoA mutase.

Liver-targeted hLB-001 is a novel gene editing therapy designed to provide durable hepatocyte expression of the *MMUT* gene without the use of exogenous promoters or nucleases [11]. hLB-001 utilizes the recombinant adeno-associated virus (AAV)-LK03 capsid, which has up to 100-fold higher transduction efficiency of human hepatocytes than AAV8 [12]. The AAV-LK03 capsid directs delivery of the genetic sequence to the nuclei of hepatocytes (Fig. 1) [12]. The genetic sequence encodes a functional copy of the human *MMUT* coding sequence flanked by 5′ and 3′ homology guides to mediate nondisruptive integration into the 3′ end of the albumin (*ALB*) locus of hepatocytes using the cell’s homologous recombination machinery [11, 13]. The genetic sequence does not include a promoter; therefore, transgene expression is dependent on precise integration into the targeted 3′ site of *ALB* to be driven by the endogenous *ALB* promoter [13]. Prior studies have demonstrated that this nuclease-free approach minimizes undesirable integration events, and results in stable expression of the transgene only when a targeted integration event occurs [13]. However, because of the low integration rate associated with in vivo homologous recombination, in vivo expansion of corrected cells is needed to achieve therapeutic levels of expression [10]. Genomic integration of *MMUT* aims to overcome some of the limitations of traditional gene therapies, such as transgene silencing due to promoter shutoff/DNA methylation or long-term dilution of episomal (i.e., nonintegrated) DNA due to rapid hepatic growth during the neonatal/pediatric period [11, 14]. Furthermore, the lack of an exogenous promoter limits the likelihood of transcription in the case of off-target integration.

*ALB* is highly expressed in the liver, allowing high levels of MMUT protein production in transduced hepatocytes [11, 15]. The hLB-001 genetic construct used here includes a peptide sequence, P2A, that induces ribosome skipping with approximately 94% cleavage efficiency to yield in-frame translation of ALB and MMUT as two separate proteins [11, 15, 16]. The 3′ end of *ALB* is tagged with the P2A sequence generating a nonnative 2A-tagged circulating protein, albumin-2A (ALB-2A) [11] that indicates gene editing has occurred. Serum levels of ALB-2A also act as a surrogate biomarker for the expression of transgenic MMUT protein since they are cotranslated.

In an MMA mouse model treated with AAV-ALB-2A-MMUT (2.5 × 10^11^ or 2.5 × 10^12^ vg/pup at birth), analysis of genomic DNA from liver samples demonstrated proper integration of the vector into the *ALB* locus, with an increase in copies detected over time suggesting expansion of corrected hepatocytes [10]. Dose-dependent increases in transgenic MMUT protein were detected in liver 3 months posttreatment. Integration and expression of the transgene were more robust in mice with MMA compared with treated wild-type animals, demonstrating a selective growth advantage over unedited MMUT-deficient hepatocytes. Transgenic MMUT protein restored enzymatic activity as demonstrated by decreases in the biomarkers methylmalonic acid and FGF21, increases in propionate oxidation, and improved survival relative to untreated controls. This study also demonstrated that the ALB-2A reporter could be detected in plasma. Plasma ALB-2A concentrations increased over time and correlated with genomic integration (*r* = 0.995; *p* < 0.0001) and with transgenic MMUT protein levels (*r* = 0.812; *p* = 0.0001). This established the ability to utilize ALB-2A as a surrogate biomarker for expression of transgenic MMUT protein, which remains within the mitochondria of transduced hepatocytes and is not secreted. A subsequent study in the same mouse model evaluated the impacts of other dose levels of AAV-ALB-2A-MMUT (2.5 × 10^13^, 5.0 × 10^13^, and 1.0 × 10^14^ vg/kg of animal) in response to a protein challenge designed to mimic metabolic stress experienced by patients with MMA [11]. AAV-ALB-2A-MMUT treatment improved survival, weight, and biomarker levels (e.g., circulating methylmalonic acid) and was accompanied by site-specific genomic integration in hepatocytes, transcription of fused *ALB-2A-MMUT* mRNA, ALB-2A protein circulation, and functional MMUT activity. The minimum efficacious dose was determined to be 5.0 × 10^13^ vg/kg, and this was selected as the starting dose for clinical trials of hLB-001.

This report describes the first-in-human phase 1/2 trial of non-nuclease-mediated gene editing using hLB-001 in pediatric participants with MMA. We also discuss our experience and key learning points that may be widely applicable to other trials of gene editing therapies in children with severe metabolic disorders.

## Participants and Methods

### Study oversight

The study was approved by the institutional review board (IRB) at each participating study site (University of Pittsburgh [MOD20010208-002]; Vanderbilt University [201305]; Seattle Children’s Hospital [20203011]; Emory University [STUDY00002158]). All participants or their parents, guardians, or legal representatives provided written informed consent and assent. The study was performed in accordance with the current Good Clinical Practice Guideline of the International Council for Harmonisation and appropriate regulatory requirements. An independent data and safety monitoring board (DSMB) and sponsor medical monitor provided study oversight.

### Study design

This was a phase 1/2 open-label study (ClinicalTrials.gov identifier: NCT04581785) to evaluate the safety, tolerability, biologic activity, and clinical efficacy of hLB-001 in pediatric participants with methylmalonic acidemia (MMA). The study consisted of a screening period, a ≥ 16-day run-in period from start of screening period to hLB-001 dosing (day 1) to establish a baseline for methylmalonic acid levels and demonstrate clinical stability, a hospitalization period, and a follow-up period of 52 weeks. Participants were eligible to enter a long-term follow-up study (ClinicalTrials.gov identifier: NCT05506254). The study is no longer enrolling.

Two dose levels of hLB-001 were planned for administration as single peripheral intravenous (IV) doses in 2 cohorts: Cohort 1 (5.0 × 10^13^ vg/kg) and Cohort 2 (1.0 × 10^14^ vg/kg). Each cohort was to consist of two parts: part A for enrollment of participants aged 3–12 years and part B for enrollment of participants aged six months to two years. After two participants developed thrombotic microangiopathy (TMA), Cohort 1 part C was added to the protocol to enroll participants aged 6 months to 12 years for further safety evaluation prior to enrolling participants at the higher dose level in Cohort 2.

For Cohort 1 parts A and B, a minimum of six weeks of postdosing safety-related data were evaluated by the independent DSMB chair and the sponsor medical monitor after the dosing of each participant. The first participant had to be at least midway through the planned corticosteroid tapering period for the safety review to be completed. The same criteria were applied after the second participant in each study part was dosed.

A full list of protocol amendments is provided in the Supplementary Information.

### Participants

Participants aged six months to 12 years at the time of dosing were eligible. Participants were required to be medically stable for the two months prior to the start of screening and have a diagnosis of severe MMA meeting all of the following criteria: isolated MMA with genetically confirmed pathogenic variants in *MMUT;* screening serum/plasma methylmalonic acid level of > 100 μmol/L with estimated glomerular filtration rate ≥ 60 mL/min/1.73 m^2^; and one or more of the following considered by the investigator to be MMA related: an unscheduled emergency room visit, hospitalization, or requirement for sick day diet in the year prior to screening visit; developmental delay, movement disorder, optic neuropathy, or feeding disorder with tube feeding requirement.

Participants were excluded if they had an organic acidemia other than isolated MMA or any other cause of hyperammonemia; had received MMA-targeted gene therapy or nucleic acid therapy; were receiving insulin or high-dose hydroxocobalamin (i.e., > 1 mg/day parenteral); received a kidney or liver transplantation, including hepatocyte cell therapy; had an estimated glomerular filtration rate of < 60 mL/min/1.73 m^2^ based on age-appropriate equations, or ongoing dialysis for renal disease; or had tested positive for anti-recombinant adeno-associated virus LK03 (AAV-LK03)-neutralizing antibodies (i.e., titers above 1:10 threshold via cell-based neutralizing antibody assay).

Participants described in this manuscript were enrolled under protocol versions 3.2, 4.0, and 5.0.

### Interventions

hLB-001 is a genetically engineered, liver-targeted, recombinant AAV-LK03 containing 5′ and 3′ DNA homology arms corresponding to the human *ALB* locus and a codon-optimized form of the human *MMUT* sequence preceded by a P2A coding sequence (see Supplementary Information for full sequence). hLB-001 was prepared as a sterile, nonpyrogenic solution stored at -65 °C or below until ready to be thawed for use and infused via filled syringes using an infusion pump. Participants received a single IV infusion over one hour on study day 1.

As AAV administration to pediatric patients has been associated with clinically significant liver enzyme elevations, prophylactic steroid administration for a minimum duration of one month is recommended [17]. Therefore, a standardized regimen of prednisolone 1.0 mg/kg/day or its equivalent (not to exceed 60 mg/day) was initiated during hospitalization 24 ± 4 h prior to starting the hLB-001 infusion. Daily corticosteroid dosing continued for a planned 30-day course followed by a 26-day taper; the course could be extended if liver enzymes remained elevated per protocol.

### Clinical assessments

Safety evaluations were performed throughout the study and included laboratory tests, vital signs, physical examinations, and electrocardiograms. Treatment-emergent adverse events (TEAEs) and treatment-emergent severe adverse events (TESAEs) were coded using the latest version of Medical Dictionary for Regulatory Activities and graded according to the National Cancer Institute Common Terminology Criteria for Adverse Events Version 5.0. Pharmacodynamic (PD) biomarkers, exploratory biomarkers, viral shedding, and antibody responses to hLB-001, MMUT, and ALB-2A were evaluated at protocol-defined time points postdosing. Liver ultrasound and alpha-fetoprotein levels were assessed at screening and at the week 52 (end-of-study) visit. Given the young age of the participants, posttreatment liver biopsies were not required, a decision that was reviewed and approved by all four IRBs overseeing the study.

### Study endpoints

Primary endpoints were the incidence of TEAEs or TESAEs and the incidence of infusion toxicities (hLB-001-related adverse events that limit, delay, or require medical intervention during administration). Secondary endpoints were change from predose baseline or average predose baseline in serum ALB-2A, serum methylmalonic acid (sMMA), serum methylcitric acid (sMCA), serum FGF21, and exhaled ^13^CO_2_ production measured by the propionate oxidation analysis method, as well as survival at one year postdose. Exploratory endpoints included evaluation of hLB-001 viral shedding and evaluation of immunologic changes. Additional exploratory endpoints can be found in the Supplementary Information.

### Assays

All laboratory tests in this study were performed at Clinical Laboratory Improvement Amendments (CLIA)-certified facilities, ensuring compliance with federal quality standards for clinical testing. During screening, evaluation of anti-recombinant AAV-LK03-neutralizing antibody titer was performed at a central laboratory using a cell-based neutralizing antibody assay (Absorption Systems/Pharmaron, Woburn, MA, USA). Quantitation of serum ALB-2A was performed by a central laboratory using an enzyme-linked immunosorbent assay (Immunologix Laboratories, Tampa, FL, USA). Quantitation of sMMA and sMCA was performed by a central laboratory using liquid chromatography tandem mass spectrometry (Mayo Validation Support Services, Rochester, MN, USA). Levels of serum FGF21 were centrally evaluated using the HMPC108 microsphere-based assay analyzed on the Luminex platform (Rules-Based Medicine [an IQVIA business], Austin, TX, USA). Breath samples were collected from participants over time after administering a ¹³C-labeled substrate ([1-¹³C]-propionate) for the analysis of ^13^CO_2_. Total CO₂ production rate (VCO_2_) was estimated as a constant based on participant body surface area (BSA):

$${{{{\rm{VCO}}}}}_{2}=5 \, {{{\rm{mmol}}}}/\min /{{{{\rm{m}}}}}^{2}\times {{{\rm{BSA}}}}\left({{{{\rm{m}}}}}^{2}\right)\times 60\min /{{{\rm{h}}}}$$

The Sercon ABCA2 gas isotope ratio mass spectrometer was used to measure the ratio of ¹³CO₂ to ¹²CO₂ in each breath sample by Metabolic Solutions, Inc. (Nashua, NH, USA). Data were analyzed by Medpace (Cincinnati, OH, USA). Breath enrichment due solely to the labeled dose above baseline was calculated and expressed as atomic percent excess (APE), representing the additional ¹³C appearing in breath CO₂ because of oxidation of the labeled substrate (expressed as a fraction). Because [1-¹³C]-propionate contains a single labeled carbon, the administered tracer dose was as mmol of ^13^C:

$${\scriptstyle{13}\atop} \! C \; {{\rm{dose}}} \; {{\rm{administered}}}({{\rm{mmol}}}) =\frac{{{\rm{dose}}}\,{{\rm{of}}}[{1{\mbox{-}}}^{13}{{\rm{C}}}]{\mbox{-}}{{\rm{propionate}}} \, \left({{\rm{mg}}}\right)}{{{{\rm{molecular}}}} \; {{{\rm{weight}}}} \; {{{\rm{of}}}} \; {{{\rm{propionate}}}}}$$

The percentage of the administered dose oxidized to CO₂ per hour (%Dose/h) was calculated as the product of breath ¹³C enrichment (APE) and CO₂ production rate (VCO_2_), normalized to the administered ^13^C dose and multiplied by 100:

$$\% \frac{{{\rm{Dose}}}}{{{\rm{h}}}}=\frac{{{\rm{APE}}}\times {{VCO}}_{2}}{{}^{13}C \; {{\rm{dose}}} \; {{\rm{administered}}}}\times 100 \%$$

Viral shedding was assessed centrally via quantitative polymerase chain reaction of participant saliva, stool, and urine to detect human *MMUT* (Charles River Laboratories, Inc., Mattawan, MI, USA). Immunologic changes were assessed through antibody or cellular immune response to recombinant AAV-LK03 or to proteins expressed following integration (ALB-2A, MMUT) endpoints. Anti-AAV2 (intact particle) mouse monoclonal, A20 antibody (Cat. No: 61055, Progen Biotech Inc., Wayne, PA, USA) was used to detect recombinant AAV-LK03, and anti-MUT polyclonal antibody (Cat. No: 17034-1-AP, Proteintech Group, Inc., Rosemont, IL, USA) was used to detect MMUT. Antibodies specific for ALB-2A (clone 57C2-2) were produced by LogicBio (Lexington, MA, USA).

### Statistical analysis

The planned sample size was approximately 8–12 participants, based on practical considerations for an ultrarare disease population. The safety population included all participants who received hLB-001. The intent-to-treat population included all participants who received hLB-001 and had baseline data and ≥ 1 postdose measurement.

No statistical hypotheses were evaluated with respect to the study endpoints. Each participant served as their own control. All analyses were descriptive. Descriptive statistics on continuous data included means, medians, standard deviations, and ranges. Categorical data were summarized using frequency counts and percentages.

Extrapolation of serum ALB-2A levels to reflect the expected necessary number of MMUT-producing cells required to provide a therapeutic benefit were modeled using published [10, 11] and de novo preclinical data.

## Results

### Participants

**Fig. 2:**
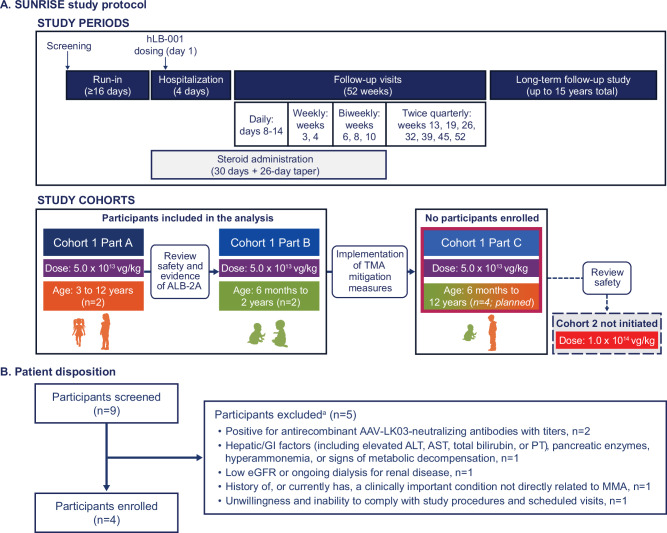
SUNRISE study protocol and patient disposition. **A** SUNRISE consisted of a screening period, a ≥ 16-day run-in period from start of screening period to single IV hLB-001 dosing, a hospitalization period starting at least one day prior to dosing and continuing for three days after dosing, and a follow-up period of 52 weeks. Participants were eligible to enter a long-term follow-up study (ClinicalTrials.gov identifier: NCT05506254). Two dose levels of hLB-001 were planned to be administered across two cohorts: Cohort 1 (5.0 × 10^13^ vg/kg) and Cohort 2 (1.0 × 10^14^ vg/kg). Cohort 1 consisted of two parts: part A enrolled participants aged 3 to 12 years, and part B enrolled participants aged 6 months to 2 years. After two participants developed TMA, Cohort 1 part C was added to the protocol to enroll participants aged 6 months to 12 years for further safety evaluation prior to enrolling participants at the higher dose level (Cohort 2). No participants were enrolled in part C, and thus Cohort 2 was not initiated. **B** Nine participants were screened, and four participants were enrolled. ^a^Participants could have met multiple exclusion criteria. AAV adeno-associated virus, ALB-2A albumin-2A, ALT alanine transaminase, AST aspartate aminotransferase, eGFR estimated glomerular filtration rate, GI gastrointestinal, IV intravenous, MMA methylmalonic acidemia, PT prothrombin time, TMA thrombotic microangiopathy.

The study was conducted at four sites in the United States (first patient enrolled April 9, 2021; last patient enrolled October 22, 2021; data cutoff date May 23, 2023). Of nine participants screened, five were excluded due to testing positive for anti-recombinant AAV-LK03-neutralizing antibodies, hepatic/gastrointestinal factors, low estimated glomerular filtration rate/ongoing dialysis, history of/current condition not related to MMA, and unwillingness to comply with study procedures (Fig. 2). Ultimately, four participants were enrolled in the study and received hLB-001 in Cohort 1, including two participants in part A and two participants in part B of the protocol (Fig. 2). No participants were enrolled in part C and thus Cohort 2 was not initiated. All four participants were included in the intent-to-treat and safety populations, and all completed the 52-week follow-up period and entered a separate, ongoing long-term follow-up study. Data are reported based on a clinical database lock date of May 23, 2023, and include two years of posttreatment data. Baseline characteristics are shown in Table 1. Participants 1, 3, and 4 were diagnosed by newborn screening. All study participants carried at least one early truncating variant in *MMUT*, either a nonsense mutation, an early frameshift leading to premature termination, or a known null allele (Table 1) [18]. At screening, Participant 1 exhibited the lowest sMMA level (79.31 µM), while Participant 3 had the lowest sMMA and serum FGF21 levels immediately prior to hLB-001 infusion (130.06 µM and 0.3 ng/mL, respectively). Participant 3 would be classified as *mut*^*–*^ MMA based on the presence of an extensively studied and prototypical *mut*^*–*^ mutation (c.2150 G > T; p.Gly717Val) in trans to a frameshift stop null allele (Table 1) [18–20]. This participant met the prespecified study screening criterion of sMMA > 100 µM in their pre-hLB-001 infusion sample. Additional details of participant medical history are provided in Supplementary Information.

**Table 1:** Baseline demographics and participant characteristics.

	Participants with MMA (*n* = 4)
Participant ages in months at dosing^a^	20, 25, 78, 114
Participant genotype (*mut* class)^b^, n (%)	
c.682 C > T; p.Arg228* (*mut*^0^); c.689 C > T; p.Thr230lle (likely *mut*^0^)^c^	1 (25)
c.322 C > T; pArg108Cys (*mut*^0^); c.671_678dupAATTTATG; p.Val227Asnfs*16 (ND)	1 (25)
c.383del; p.Lys128Argfs*52 (ND); c.2150 G > T; p.Gly717Val (*mut*^*–*^)	1 (25)
c.1207 C > T; p.Arg403* (*mut*^0^); c.278 G > A; p.Arg93His (*mut*^0^)	1 (25)
Participant sex, n (%)	
Female	1 (25)
Male	3 (75)
Participant race, n (%)	
White	2 (50)
Other	2 (50)
Participant weights in kg at baseline^a^	11.2, 13.5, 19.2, 29.3
Participant average predose serum methylmalonic acid level in µmol/L^a^	123.5, 302.5, 666.96, 689.6
Participant receiving levocarnitine treatment, n (%)	4 (100)
Participant receiving carglumic acid, n (%)	2 (50)

^a^Due to the rarity of gene editing studies involving pediatric participants with MMA, and to protect participant identity and maintain confidentiality, we minimized the inclusion of identifiable individual-level data, such as exact age and weight. Accordingly, individual values are reported in order from lowest to highest, rather than by enrollment sequence. ^b^Confirmatory molecular genetic tests for each participant were completed in CLIA-certified labs: Participants 1 and 4 - Invitae, San Francisco, CA, USA; Participant 2 - Prevention Genetics, Marshfield, WI, USA; Participant 3 - Greenwood Genetic Center, Greenwood, SC, USA. *mut* class determined according to Forny et al. (2016) [18]. See also additional details in Fig. 4 inset table. ^c^A mutation at the same base, c.689 C > G; p.Thr230Arg, is classified as *mut*^0^ by Forny et al. (2016) [18]. See also Fig. 4 footnotes.

*MMA* methylmalonic acidemia, *sMMA* serum methylmalonic acid, *ND* not defined.

### hLB-001 dosing

All participants received the planned hLB-001 dose of 5.0 × 10^13^ vg/kg via intravenous (IV) infusion. The median (min, max) total dose in the four participants was 8.5 × 10^14^ (5.0 × 10^14^, 1.4 × 10^15^) vg. Duration of hospital stay, including day of hLB-001 infusion, was four days for Participant 1, five days for Participants 2 and 4, and six days for Participant 3. Participants 1, 2, and 4 were admitted one day prior to hLB-001 infusion; Participant 3 received an infusion three days after admission because of a two-day delay due to symptoms of upper respiratory tract infection. Corticosteroids (prednisolone 1.0 mg/kg/day, not to exceed 60 mg/day) were administered to all participants starting one day prior to the hLB-001 infusion (day -1). In addition, Participant 4 also received a course of methylprednisolone from day 8 to day 23. The median (min, max) number of days on corticosteroids for all participants was 79.5 (55, 127), and number of days on full-dose corticosteroids was 48.5 (30, 73). All participants were on a dietary management plan, meeting the standard practice guidelines for individuals with MMA [6, 21].

### Safety and tolerability

All participants experienced transient increases in liver chemistry tests based on laboratory values (Supplementary Fig. 1). Elevated alanine transaminase (ALT) and aspartate aminotransferase (AST) remained < 2× the upper limit of normal (ULN) in Participants 1 and 3 and did not necessitate increasing the length of prednisolone administration. For Participants 2 and 4, increases in ALT resulted in the need to extend the steroid course, including time on full-dose steroids. ALT levels for Participant 2 peaked at 2.3× ULN, and levels for Participant 4 exceeded 8× ULN. Changes from baseline in height and weight of all participants are shown in Supplementary Fig. 2. No notable adverse events related to corticosteroid therapy were reported. All participants were alive at 1 year (secondary endpoint) and at the two-year follow-up.

All hLB-001 infusions were completed without interruption. All four study participants experienced at least one TEAE (Table 2), but no TEAEs led to dose interruption or study withdrawal. Cytokine release syndrome, headache, increased transaminases, nausea, pyrexia, thrombotic microangiopathy (TMA), and vomiting were considered related to hLB-001.

**Table 2:** TEAEs and TESAEs.

System organ class preferred term, n (%)	Part A (3-12 years) *n* = 2	Part B (6–36 months) *n* = 2	Overall *n* = 4
**Participants with at least 1 TEAE**	2 (100)	2 (100)	4 (100)
Participants with at least 1 TESAE (events in **bold**)	1 (50)	2 (100)	3 (75)
**Metabolism and nutritional disorders**			
Decreased appetite	1 (50)	1 (50)	2 (50)
Dehydration	0	1 (50)	1 (25)
Hyperglycemia	0	1 (50)	1 (25)
Hyperkalemia	**1 (50)**^a^	0	1 (25)
**Gastrointestinal disorders**			
Vomiting	1 (50)^b^	1 (50)^b^	2 (50)
Diarrhea	0	1 (50)	1 (25)
Nausea	1 (50)^b^	0	1 (25)
**Immune system disorders**			
Cytokine release syndrome^c^	1 (50)^b^	**1 (50)**^b^	2 (50)
Seasonal allergy	0	1 (50)	1 (25)
**Blood and lymphatic system disorders**			
Thrombotic microangiopathy (TMA)	0	**2 (100)**^d^	2 (50)
**General disorders and administration site conditions**			
Pyrexia	1 (50)^b^	1 (50)^b^	2 (50)
Fatigue	1 (50)	0	1 (25)
**Infections and infestations**			
Asymptomatic COVID-19	1 (50)	0	1 (25)
Respiratory tract infection viral	0	1 (50)	1 (25)
Viral infection	0	1 (50)	1 (25)
**Nervous system disorders**			
Lethargy	1 (50)	1 (50)	2 (50)
Headache	1 (50)^b^	0	1 (25)
**Ear and labyrinth disorders**			
Deafness neurosensory	0	1 (50)	1 (25)
**Investigations**			
Transaminases increased^e^	0	1 (50)^b^	1 (25)
**Procedures performed during treatment of TMA**^**f**^			
Eculizumab treatment	0	1 (50)	1 (25)
Hemodialysis	0	1 (50)	1 (25)
Intubation and mechanical ventilation	0	1 (50)	1 (25)
Intravenous fluids and parenteral nutrition	0	1 (50)	1 (25)
Transfusion (packed red blood cells; platelets)	0	1 (50)	1 (25)

TEAEs and TESAEs were coded using the latest version of MedDRA and graded according to the CTCAE version 5.0. Adverse events were identified and reported by individual investigators. Any participant with multiple events in one System Organ Class (SOC) or Preferred Term (PT) was counted only once for that SOC or PT, using the event with the strongest relationship to study drug. Events were considered unrelated to hLB-001 unless noted.

^a^Participant 2 had two events of hyperkalemia that were considered TESAEs (indicated by **bold**). Events occurred on day 3 (potassium, 6.5 mmol/L [reference range: 3.5–5.5]; glucose 4.33 mM [reference range: 3.33–5.83]) and day 289 (potassium, 6.8 mmol/L; glucose, not collected). Events resolved on days 4 and 303, respectively. ^b^Considered related to hLB-001. ^c^For both participants, the verbatim term reported by investigators was “cytokine release syndrome,” which maps to the MedDRA preferred term, characterized by fever, tachypnea, headache, tachycardia, hypotension, rash, and/or hypoxia caused by the release of cytokines. ^d^Considered related (Participants 3 and 4) to hLB-001. ^e^All participants experienced transient increases in liver function tests based on laboratory values (Supplementary Fig. 1). ^f^Not a formal MedDRA classification.

*CTCAE* common terminology criteria for adverse events, *MedDRA* Medical Dictionary for Regulatory Activities, *TEAE* treatment-emergent adverse event, *TESAE* treatment-emergent serious adverse event.

Participants 2, 3 and 4 experienced events considered TESAEs, all of which resolved during the study. Participant 2 (part A) experienced two TESAEs of hyperkalemia, which is known to occur in association with MMA [22, 23]. The first event occurred three days after hLB-001 infusion and was considered severe and unrelated to hLB-001. The event resolved by four days after infusion. The second event occurred 289 days after infusion, was of moderate severity, and was considered unlikely to be treatment related. The event resolved without intervention after 13 days. Two days after hLB-001 infusion, Participant 4 (part B) experienced a TESAE of cytokine release syndrome (mild severity) that was considered treatment related but resolved by day 3 without medical intervention, and the participant was discharged.

Two participants in part B experienced TESAEs of TMA. TMA was noted in Participant 3 at the two-week visit per protocol (12 days after hLB-001 infusion), with vomiting and fever (noted by the family) two and three days prior to the visit, respectively. The participant presented with microscopic hematuria but otherwise appeared well at the visit. Laboratory tests indicated renal dysfunction (elevated creatinine, hematuria, and proteinuria) and evidence of hemolysis (decreased hemoglobin, reticulocytosis at 3.0x ULN, and elevated lactate dehydrogenase) but no thrombocytopenia. Subsequent testing revealed elevated soluble C5b9 levels, indicating complement system activation. The TMA event was considered severe and related to hLB-001. However, the participant recovered with supportive care (IV fluids through day 16 after hLB-001 infusion and parenteral nutrition only) and did not require hemodialysis or treatment with eculizumab. The participant was discharged six days after admission to the hospital (18 days after hLB-001 infusion). No recurrence has been reported as of the cutoff date.

In Participant 4, TMA was first noted six days after hLB-001 infusion while at home by urine dipstick showing trace blood and protein. Evidence of complement-mediated process resulting in renal dysfunction, anemia, and thrombocytopenia was observed. The event was considered severe and life-threatening and hLB-001 related. Participant 4 was admitted to the pediatric intensive care unit six days after hLB-001 infusion and required eculizumab treatment (600 mg eight days after hLB-001 infusion and 300 mg 15 days after infusion), intermittent hemodialysis (nine total courses between 10 and 18 days after hLB-001 infusion), mechanical ventilation due to respiratory insufficiency from fluid overload/retention caused by acute kidney failure secondary to TMA/atypical hemolytic uremic syndrome (aHUS) (10–14 days after hLB-001 infusion), and transfusions of packed red blood cells (9, 10, and 17 days after hLB-001 infusion) and platelets (10 days after hLB-001 infusion). The participant recovered and was discharged 21 days after admission (27 days after hLB-001 infusion). As part of eculizumab treatment, the participant also received the full schedule of childhood vaccinations, including the meningococcal vaccine. Some were administered during the inpatient stay, with the remainder provided during outpatient follow-up. No recurrence has been reported as of the cutoff date.

### Genetic testing for TMA risk factors

Genetic testing for TMA risk factors was performed in three participants after treatment (Supplementary Table 1). A TMA genetic testing panel for Participant 2 identified alterations in genes associated with aHUS, including *C3AR1* (variant of unknown significance [VUS]), *C2* (likely a benign variant), and *MCP/CD46* (several polymorphisms). The TMA-specific panel for Participant 3 identified alterations in aHUS-associated genes: *ADAMTS13* (benign polymorphism), *DGKE* (VUS), *CFI* (likely a benign variant), *CFH* (several polymorphisms), and *MCP/CD46* (one polymorphism). Because of history of thrombosis following prior central venous catheterization, Participant 4 had been previously tested for two genetic variants associated with increased risk of thrombosis, Factor V Leiden and a prothrombin II variant (G20210A), but neither were detected. As part of Participant 4’s TESAE management during the hospital pediatric intensive care unit admission, an aHUS genetic panel testing was performed and identified a heterozygous variant of uncertain significance in *MCP/CD46* previously associated with aHUS [24, 25].

### Serum ALB-2A levels

**Fig. 3:**
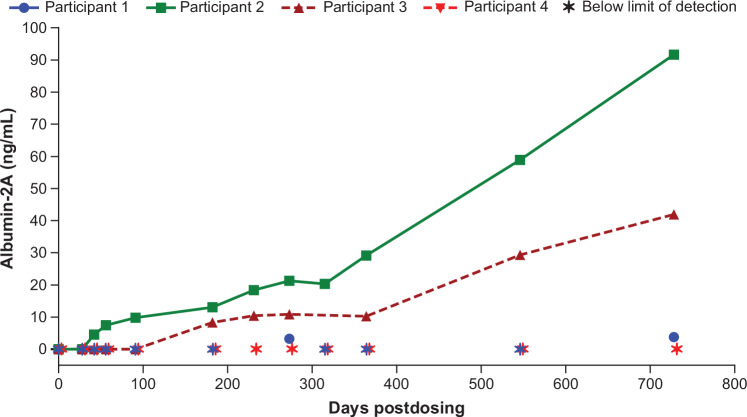
Serum ALB-2A levels. Serum levels for the surrogate biomarker ALB-2A, demonstrating targeted integration of corrective *MMUT* in participants. Participant 4 had two samples with detectable levels of ALB-2A (raw mean values of 0.74 ng/mL and 1.95 ng/mL at days 42 and 56, respectively). The lower limit of quantification for the assay was 2.44 ng/mL. As of the data cutoff date (May 23, 2023), serum ALB-2A levels remained below the range expected to provide a therapeutic benefit (1000–3000 ng/mL) based on extrapolation from mouse models [10, 11]. ALB-2A, albumin-2A.

All participants showed measurable serum levels of ALB-2A (Fig. 3). This surrogate biomarker provides evidence of targeted integration of the genetic sequence, including the corrective *MMUT* transgene. Although Participant 4 had detectable ALB-2A (raw mean values of 0.74 ng/mL and 1.95 ng/mL at days 42 and 56, respectively), the levels were below the lower limit of quantification for the assay (2.44 ng/mL). Serum ALB-2A levels were below the limit of quantification for Participant 1 for all but two time points. For Participants 2 and 3, levels of ALB-2A increased from 4.6 ng/mL (day 41) and 6.9 ng/mL (day 133), respectively, to 91.6 ng/mL and 41.9 ng/mL at the two-year time point (≈ day 728), suggesting continued expansion of edited hepatocytes. However, as of the data cutoff date (May 23, 2023), serum ALB-2A levels remained below the range expected to provide a therapeutic benefit (1000–3000 ng/mL) based on extrapolation from mouse models [10, 11].

### Other pharmacodynamic biomarkers

**Fig. 4:**
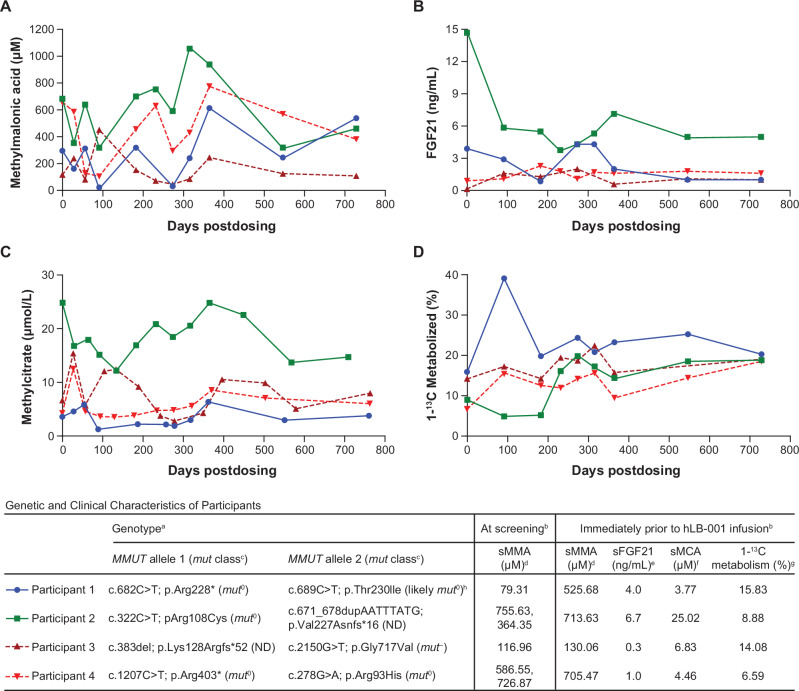
Levels of other pharmacodynamic biomarkers. During the two-year study period, levels of **A** sMMA, **B** serum FGF21, **C** sMCA, and **D** propionate oxidation did not reach levels associated with clinically meaningful therapeutic benefit in any participants. ^a^Confirmatory molecular genetic tests for each participant were completed in CLIA-certified labs: Participants 1 and 4 - Invitae, San Francisco, CA, USA; Participant 2 - Prevention Genetics, Marshfield, WI, USA; Participant 3 - Greenwood Genetic Center, Greenwood, SC, USA. ^b^Data in graphs slightly differ from the table; day 0 data point in graphs represent average of values obtained during screening and immediately before hLB-001 infusion (not all screening values shown in table). ^c^According to Forny et al. (2016) [18]. Patients with MMA subtypes *mut*^*0*^ and *mut*^*–*^ have: sMMA levels ranging from < 10 to > 1000 µM [34]; sMCA levels > 1.0 µM (dried blood spot and plasma) [61–63]; FGF21 levels ranging from several hundred to several thousand pg/mL, depending on disease severity and organ involvement [34, 64, 65]; and propionate oxidation from measurement of exhaled CO_2_ production in 120 min ≈15–45% [34]. ^d^Reference range in healthy children (1 to ≤ 11 years): ≤ 0.30 µM [66]. ^e^FGF21 > 1.5 ng/mL is associated with long-term complications in patients with MMA [64]; the protocol-defined target was ≤ 1.5 ng/mL at 1 year following hLB-001 administration. ^f^Reference range: ≤ 0.1 µM (Mayo Validation Support Services, Rochester, MN, USA). ^g^Reference range in healthy volunteers: ≈35–55% [34]. ^h^A mutation at the same base, c.689 C > G; p.Thr230Arg, is classified as *mut*^0^ by Forny et al. (2016) [18]. The p.Thr230Arg variant replaces a polar threonine with a bulky, positively charged arginine, whereas p.Thr230Ile replaces threonine with a bulky, nonpolar isoleucine; both substitutions are predicted by CUPSAT (https://cupsat.brenda-enzymes.org/ using PDB: 8DYL) to have similarly unfavorable torsional effects. The basis for Participant 1’s low ( < 100 µM) screening sMMA despite more severe pre-hLB-001 infusion biomarker profiles remains unclear. CLIA Clinical Laboratory Improvement Amendments, sFGF21 serum fibroblast growth factor 21, sMCA serum 2-methylcitric acid, sMMA serum methylmalonic acid, MMUT methylmalonyl-CoA mutase gene, ND not defined.

During the two-year study period, sMMA, serum FGF21, sMCA, and propionate oxidation did not reach levels associated with clinically meaningful therapeutic benefit in any participants (Fig. 4). Additional laboratory data collected (data not shown) included measurements of ketones (urine dipstick), pancreatic enzymes (blood amylase and lipase), blood albumin, and plasma total carnitine.

### Viral shedding and immune response

hLB-001 viral shedding (exploratory endpoint) was detected in all participants starting on day 7 after dosing (first assessment) in saliva, urine, and stool. Viral shedding decreased over time and was undetectable starting at week 39 and/or 52 in the participants with samples at those time points (Supplementary Fig. 3). Viral particles were highest in urine and lowest in saliva. Antibody responses to ALB-2A or MMUT (exploratory endpoint) were not detected in any of the participants during the study. Outcomes for antibody responses to ALB-2A or MMUT were binary (“negative”/”positive”), and all responses were “negative” at all time points tested. However, all participants had detectable antibodies against recombinant AAV-LK03 (exploratory endpoint) starting at the first assessment (at week 8, 13, or 26, depending on the individual) that persisted through the last assessment (week 52).

### Study termination

Enrollment in the study was terminated due to initial evidence of lack of clinical efficacy, associated with lower-than-expected serum ALB-2A levels and biomarker response. All four participants who had already been dosed were monitored for safety and clinical efficacy through their scheduled end-of-study visit.

## Discussion

MMA is a devastating disease affecting individuals early in life, and no curative treatments are available. Due to the genetic basis of MMA, gene editing therapy represents an attractive alternative to liver transplantation. Conducting trials in MMA and similar rare diseases with fragile pediatric participants, especially with gene editing therapies, has numerous challenges [26–28].

We conducted the first-in-human trial of gene editing therapy in MMA, which enrolled young pediatric participants. Our study provides proof-of-concept that liver-targeted gene editing using the AAV-LK03 capsid and targeting the endogenous *ALB* locus without the use of nucleases can genetically alter human hepatocytes. Among all four participants, a single hLB-001 infusion resulted in evidence of integration as indicated by detection of the tagged, nonnative ALB-2A protein in participant blood samples. ALB-2A expression serves as a surrogate for MMUT expression as direct evaluation (e.g., liver biopsy) was not feasible. Participants 2 and 3 showed increased levels of the ALB-2A biomarker over time indicating stable, genomic integration and target gene expression, but these did not reach the level of therapeutic benefit during the two-year follow-up period. Participants 1 and 4 did not have sustained increases in ALB-2A levels. While published reference or target ranges for other biomarkers (e.g., sMCA, serum FGF21) served as comparators (see Fig. 4 footnotes), the broader assessment of clinical benefit remains limited by the scope of biomarkers evaluated and the study design.

We have several hypotheses for these observations. Low integration was expected because in an effort to improve safety, a nuclease-based gene editing approach was not used. Integration was dependent on homologous recombination [10], which is a rare event especially in quiescent hepatocytes rather than those in S phase of the cell cycle [29, 30]. The loss of ALB-2A expression in Participant 4 was temporally associated with elevation of transaminases and thus could have been due to in situ elimination of corrected hepatocytes via an inflammatory response. Furthermore, the ability of and timeline for hLB-001-corrected hepatocytes to expand in patients with MMA is currently unknown. Factors potentially affecting this expansion could be an individual’s age, disease severity, genetic background, and disease or treatment courses following gene editing, or other factors. In the case of Participant 1, it is possible that this participant’s disease was sufficiently mild and controlled, reducing selective pressure for the edited hepatocytes. In contrast, Participant 2 had high levels of MMA-associated biomarkers (e.g., methylmalonic acid, FGF21, methylcitrate) at baseline indicating high disease burden, which may have increased selective pressure for edited hepatocytes, resulting in the high levels of ALB-2A (and by extension MMUT) observed. Furthermore, many patients with severe *MMUT* genotypes harboring early stop codon mutations with no detectable residual enzyme activity (*mut*^0^ classification) might be prone to a T-cell–mediated immune response to the expressed transgene product, which was not studied here. This concept has precedent in mouse models of Duchenne muscular dystrophy [31] and in patients with α-1-antitrypsin deficiency [32]. Participants 1 and 4 harbor early stop codon mutations in *MMUT* (Fig. 4 inset table) and had significantly low levels of ALB-2A (Fig. 3). However, Participants 2 and 3, with more ALB-2A (Fig. 3), harbor frameshift mutations but also another *MMUT* allele with a missense variant (Fig. 4 inset table). Participant 3, classified as an individual with *mut*^–^ MMA, has an *MMUT* prototypical allele classified as *mut*^–^, which might have allowed for maintenance of transgene expression by modulating any T-cell immune response. Lastly, the two-year follow-up period of this current analysis might be too short for sufficient expansion of corrected hepatocytes. In the *MMUT*-deficient mouse models treated with a similar AAV gene editing approach, expression of the *MMUT* gene increased from approximately 1–2% at three and six months to approximately 5% at eight to ten months and approximately 9% after 11 months [10]. Even though substantial increases in hepatic *MMUT* over time were observed in mice [10], it is possible that the rate at which transduced cells expand in hepatic tissue is species specific, and that the differences noted among the participants could reflect differences in the timing of expansion between mice and humans in addition to other factors, such as disease progression. Participants 2 and 3 exhibited continued expression of ALB-2A suggesting ongoing expansion of corrected hepatocytes through last follow-up, and as expansion continues, a therapeutic benefit might still be seen. All participants entered the long-term extension (NCT05506254); therefore, continued follow-up could reveal longer-term effects.

Levels of methylmalonic acid have been shown to fluctuate greatly in individuals with MMA [33]. Indeed, in this study, participants showed a large fluctuation of methylmalonic acid levels, sometimes having values that decreased below 100 µmol/L and other times having values higher than baseline (Fig. 4A), possibly related to current diet and/or health of participants. Fluctuations in FGF21 levels were also observed throughout the study, with no apparent trend in the data (Fig. 4B). In all participants, propionate oxidation was higher at last observation than at baseline (Fig. 4D). However, the clinical relevance of this is unclear, as levels were still much lower than those seen in individuals unaffected by MMA ( ≈ 35% to 55%) [34], and the CO_2_ production estimate used in this study (5 mmol/min/m^2^ of body surface area) may differ from actual production rates in patients with MMA.

Regarding the safety of hLB-001, all participants in the study had elevations in liver enzymes, which were expected based on other AAV-based therapies [17]. In Participants 2 and 4, extended corticosteroid use was required (Supplementary Fig. 1); however, all participants had normal ALT and AST levels at last follow-up. Although corticosteroid use in individuals with MMA is associated with risks due to catabolic effects on muscle [21], no notable corticosteroid-related adverse events were observed in this study, even with extended courses of treatment, suggesting that corticosteroids can be safely administered as needed to patients with this condition who are receiving gene editing therapy.

Studies of other AAV gene therapies have suggested that complement system activation in response to AAV can increase the risk of TMA and aHUS [35, 36], and genetic alterations associated with these conditions have been described [37–39]. Three of the four participants in our study underwent genetic testing for risk factors associated with TMA and/or aHUS after dosing. All three participants had VUS, polymorphisms, or benign variants in genes associated with aHUS, two of whom experienced TMA. Due to the limited sample size, a definitive association of TMA/aHUS-associated gene variants and an increased risk of developing these conditions cannot be concluded at this time. However, establishing molecular genetic safety measures prior to study participation to identify candidates at risk for adverse events that may be related to gene editing could inform better predosing risk/benefit counseling and institution of postdose precautionary measures.

The current study adds to the existing knowledge of AAV gene therapies. As published and reviewed elsewhere, AAV gene therapies, including liver-targeted gene-based editing approaches, have been developed to treat a wide variety of diseases, including rare diseases, such as hemophilia and spinal muscular atrophy (SMA) [40–42]. Studies of these therapies have demonstrated their efficacy and led to approvals by the US Food and Drug Administration and the European Medicines Agency [41, 42] with each new study bringing additional insights for the field. One aspect common to studies of AAV therapies is the need to identify the ideal therapeutic dose that balances risks of toxicity and benefits of efficacy [42]. Treatment with AAV gene therapy may increase the risk of hepatotoxicity (e.g., elevated liver aminotransferase), complement-related adverse events (e.g., TMA, aHUS), neurological toxicity (e.g., dorsal root ganglia [DRG] degeneration), and insertional mutagenesis [26, 35, 42, 43]. For example, hepatotoxicity has been observed in studies of liver-targeted AAVs in patients with hemophilia A (SPK-8011 at 1.5 × 10^12^ vg/kg and 2.0 × 10^12^ vg/kg; SPK-8016 at 5.0 × 10^11^ vg/kg), patients with ornithine transcarbamylase deficiency (AAVLK03hOTC at 6.0 × 10^11^ vg/kg), and patients with X-linked myotubular myopathy (resamirigene bilparvovec [AT132] at 1.3 × 10^14^ vg/kg and 3.5 × 10^14^ vg/kg) [43–47]. Regardless of AAV vector, capsid-triggered activation of the innate and adaptive immune responses can contribute to toxicities, including complement-mediated TMA/aHUS, which can be life threatening [35, 43]. These serious AEs more frequently occur with systemic delivery of high AAV vector doses, as observed in trials of Duchenne muscular dystrophy (SGT-001 at 5.0 × 10^13^ vg/kg and 2.0 × 10^14^ vg/kg; fordadistrogene movaparvovec [PF-06939926] at 2.0 ×10^14^ vg/kg), SMA (onasemnogene abeparvovec at 1.1 ×10^14^ vg/kg), Fabry disease (4D-310 at 1.0 ×10^13^ vg/kg), and Danon disease (RP-A501 at 1.1 × 10^14^ vg/kg) [35]. Although AAV-mediated oncogenesis (e.g., hepatocellular carcinoma) has been observed in animal models, no cases of genotoxicity have been reported in human clinical trials [43]. Similarly, neurological toxicities have been limited among patients receiving AAV gene therapies (e.g., two reports of DRG degeneration) [43]. Additionally, a number of deaths have been reported in patients who have received AAV-based gene therapy, including resamirigene bilparvovec [45], fordadistrogene movaparvovec [48], and onasemnogene abeparvovec [35]. Although some deaths have apparent associations with treatment-related AEs (e.g., TMA), the etiology is not always clear.

Overall, there is dose-dependent relationship between AAVs and adverse events [26, 42]. However, the range of doses used across studies and the differences in adverse events highlights the need for individualized studies of each disease/therapy to learn more about the field. Our findings suggest the dose used was insufficient to elicit a robust response in all participants, but with this dose, the incidence of adverse events was relatively low and was consistent with toxicities observed with other AAV therapies (including those using other liver-targeting capsids). Liver-targeted gene editing without the use of nucleases may not ultimately be an effective treatment for MMA, but additional investigations of nuclease-free gene editing approaches for MMA and other inborn errors of metabolism are warranted.

Although the hLB-001 trial was terminated, our experience with this trial led to important insights and can inform future trials of gene editing therapy in MMA or similar metabolic disorders. For instance, 2 of 4 participants were nonwhite (Table 1), underscoring the importance of considering ancestry in the interpretation of genetic risk particularly for TMA or aHUS. Population-based analyses of VUS in genes associated with TMA and aHUS have been conducted in nonwhite populations, most notably in Korean adults, revealing distinct, population-specific variant profiles and recurrent missense variants not observed in European datasets [49]. These findings highlight the need for ancestry-specific variant interpretation when evaluating TMA/aHUS-associated genes. There remains a paucity of large-scale, multiethnic studies, and the clinical significance of many VUS in nonwhite populations remain unclear. Many variants previously suspected of pathogenicity may, in fact, be benign polymorphisms with ancestry-driven frequency differences, further complicating interpretation. In this study, while the TMA events appear most likely attributable to AAV vector infusion, the underlying genetic predisposition to TMA/aHUS cannot be fully excluded (see legend of Supplementary Table 1). Genetic testing for TMA/aHUS-associated alleles remains complex, and in this context, the etiology of TMA remains inconclusive. Nevertheless, pretreatment genetic screening for TMA/aHUS risk variants would help clinicians and participating families better understand individualized treatment risks, support informed consent, and guide clinical decision-making, including subspecialty consultation and pre- and postinfusion monitoring. Genetic screening could consist of exome sequencing or, preferably, whole-genome sequencing, which could identify promoter, deep intronic pathogenic, or likely pathogenic variants. Again, more data beyond that of this small trial are necessary to confirm the utility of exome/genome pretrial screening approaches. Information sharing among investigators proved extremely valuable, particularly around additional evaluations, interventions, and clinical management of trial participants between sites and beyond the study protocol. Thus, close communication and clinical resource integration of the teams managing the participant is crucial.

AAV-based therapy has been associated with clinically significant liver enzyme elevations in pediatric patients, which are typically addressed using prophylactic steroid administration [17]. Due to concerns about protracted use of steroids in participants with MMA, the protocol-defined duration was 30 days at the full dose followed by a taper (unless liver function abnormalities persisted). We found that extended corticosteroid treatment beyond 30 days was manageable and, importantly, helped avoid transaminase elevation, which can reduce AAV-based therapy efficiency [50]. Immunosuppressive/anti-inflammatory regimens might also provide protection against TMA, and approaches modeled on liver transplantation could also be considered in liver-based gene editing treatments.

Some limitations were specific to our study design. Because obtaining liver biopsies was not feasible for this population, we were unable to directly confirm genomic integration of the vector construct or expression of transgenic MMUT, which is localized to the mitochondria (e.g., not detectable in serum/blood under typical circumstances). Furthermore, other means of assessing these events (e.g., analysis of circulating DNA for genomic integration) were not included in the protocol. The impact of any uncleaved proteins also remains unclear. We did not directly assess ALB-2A-MMUT, but translation of uncleaved ALB-2A-MMUT is likely rare (about 6% of events) based on estimated P2A cleavage efficiency, and in our study, no participants developed antibody responses to MMUT, suggesting no or limited aberrant secretion of MMUT or ALB-2A-MMUT.

Future gene editing therapies and studies should consider utilizing delivery of gene editing therapies directly to specific organ(s) to improve outcomes [51–53] and overcome limitations of systemic delivery, such as generalized immune response to the viral vector [54]. Further clinical benefit of gene editing therapies may be enhanced through technological improvements (e.g., modification of AAV capsids and vector genomes), alternative mechanisms of delivery (e.g., different viral vectors, nonviral vectors), and/or various base-editing technologies [55–60].

This first-in-human trial of hLB-001 gene editing therapy in MMA resulted in integration of the target gene in multiple participants without the use of nucleases. In the MMA mouse model treated with AAV-ALB-2A-MMUT, integration was demonstrated by analysis of genomic DNA from liver samples [10]; however, in this human study, it is presumed based on detection of serum ALB-2A levels. Insights from this study could guide targeted screening and intervention efforts for populations with *MMUT* mutations in future research. Two patients in the younger cohort developed TESAEs of TMA. Given the significant morbidity and mortality associated with MMA and risks associated with transplantation and immunosuppression, future trials of gene therapies for this disorder are warranted especially with approaches to increase the genome editing efficiency in humans.

## Supplementary information

Supplementary Information

## Data Availability

Alexion, AstraZeneca Rare Disease, will consider requests for disclosure of clinical study participant-level data provided that participant privacy is assured through methods, such as data deidentification, pseudonymization, or anonymization (as required by applicable law), and if such disclosure was included in the relevant study informed consent form or similar documentation. Qualified academic investigators may request participant-level clinical data and supporting documents (statistical analysis plan and protocol) pertaining to Alexion-sponsored studies. Further details regarding data availability and instructions for requesting information are available in the Alexion Clinical Trials Disclosure and Transparency Policy at https://www.alexionclinicaltrialtransparency.com/data-requests/.
