# Supplementary material for: First-in-human nuclease-free homologous recombination-dependent gene editing in pediatric patients with methylmalonic acidemia: results of a phase 1/2 study
Source: Gene Ther. 2026 Mar 30;33(3):345–57. doi: 10.1038/s41434-026-00609-1 (PMC13226049; doi:10.1038/s41434-026-00609-1)
Supplement: Supplementary file 1 — Supplementary Information [file 41434_2026_609_MOESM1_ESM.docx]

# Supplementary Information

## Participant medical history

Baseline characteristics and medical history of the four participants indicated high burden of disease.

- At baseline, all had intellectual impairment/developmental delay, three had metabolic decompensation, and three had vomiting and feeding difficulties. Additionally, one participant (Participant 2) had poor growth, vision loss (two months prior to movement disorder), and movement disorder possibly related to metabolic stroke (in basal ganglia) due to MMA, and another had hypotonia/weakness and lethargy/fatigue.
- Baseline medications included levocarnitine (n=4), carglumic acid (n=2), enoxaparin, hydroxocobalamin (Participant 1 was treated empirically with hydroxocobalamin 1000 µg once daily before genotyping, with the intent of supporting possible vitamin B12–responsive MMUT activity; subsequent genetic testing performed during this clinical trial confirmed *mut⁰* MMA), melatonin, metronidazole, sodium benzoate, sodium citrate, ubidecarenone, vitamin D (not otherwise specified) (n=1), and vitamins (not otherwise specified) (n=1).
- Notable medical history and procedures prior to study entry included gastric tube placement (n=4), central venous catheterization (n=2), deep vein thrombosis (n=2), basal ganglia stroke (n=1), nasogastric tube placement (n=1), nontunneled hemodialysis catheter site insertion (n=1), and seizures (n=1).

## Key protocol amendments

Version 1

- NA; no patients treated under this version

Version 2

- Schedule of events: updated to include additional time points for antibody response and ammonia, nutritional laboratory parameters, dietary optimization, and anti-albumin antibody

testing have been added, and the screening period has been reduced

- Section 4: survival added as secondary endpoint; multiple endpoints moved from secondary to exploratory
- Section 5.1: cohort design updated to include 2 doses of hLB-001 and age de-scalation within each cohort; sentinel patient safety review increased to 6 weeks
- Section 5.2: study suspension rules have been updated
- Section 5.3.3. and 5.3.4: Retesting criteria for screening has been updated
- Section 6.2: Inclusion criteria #5 has been updated
- Section 6.3: Exclusion criteria #6 has been updated
- Section 7.9: Details regarding dietary management have been added
- Section 10.2: Additional information regarding the use and monitoring for adverse effects due to prophylactic steroids has been added
- Throughout: Multiple clarifications and administrative corrections have been implemented

Version 3

- Schedule of events: added urine ketones to Day -1 to Day 3 and week 6
- Clarified 2-month corticosteroid administration (high-dose and taper)
- Added a maximum daily dose of corticosteroids of 60 mg
- Clarified DSMB review to focus on potential catabolic events
- Section 10.2: updated monitoring language and added instructions for tapering, potential continuation of steroids, and cortisol testing
- Updated DSMB reviews to reflect timing of safety review and steroid tapering
- Updated study suspension rules

Version 3.1

- Schedule of events: added albumin-2A at week 6; removed exploratory DNA sample
- Endpoints and section 7.15: removed exploratory DNA sample

Version 3.2

- Schedule of events and endpoints: methylcitrate added
- Endpoints: Albumin 2A moved from exploratory to secondary
- Schedule of events: removed neutralizing antibody titer at week 52

Version 4.0

- Added Saudi Arabia
- Schedule of events: added collection of height/weight to baseline visit
- Included language regarding pregnancy testing, female patients of child-bearing age

Version 5.0

- Schedule of events: removal of albumin locus DNA sample at screening
- Schedule of events: changed timing of neutralizing antibody titer from 90 days to 30 days prior to dosing out of an abundance of caution in case of seroconversion
- Removal of sick day diet measurement in inclusion criteria and in healthcare utilization, as this measure is not consistently defined across study sites
- Clarified that methylmalonic acid testing is performed via serum
- Added section 5.3.4 to allow for subject rescreening
- Updated exclusion criteria # 11: Incorporating liver function testing in the exclusion criteria of AAV-based gene therapy trials is a standard practice aimed at minimizing risk of hepatic damage and inflammation, but the levels of these measures at which the risk becomes excessive have not been established. In the absence of sufficient evidence supporting a specific approach, we performed a review of the literature and www.clinicaltrials.gov to determine common practice, which showed that studies using liver-targeted AAV vectors typically include measurements of alanine aminotransferase (ALT), aspartate aminotransferase (AST), and total bilirubin, with exclusion criteria cut-offs set at >2 or >3x the upper limit of normal (NCT 0248092, 02395342, 03306277, 02651675, 04105166). AST is present in several tissues outside the liver, including muscle, brain, pancreas, lung, kidney, RBCs, and leukocytes, and therefore is a non-specific indicator of liver disease (Botros M 2013). On the other hand, ALT is specific to the liver, and the two tests are usually highly correlated (i.e. when ALT is high, AST is as well). Therefore, mild and isolated elevations in AST typically do not indicate liver disease but rather originate from non-hepatic sources, or are an artefact caused by hemolyzed RBCs (Xu Q 2015). No adverse events or altered liver function were reported in preclinical toxicology studies of cLB-001 in juvenile non-human primates. Isolated, spontaneous, reversible and low magnitude changes in ALT were described in some animals, which were mostly <2xULN, not dose dependent, and rarely accompanied by a mild AST elevation (<2xULN). Because of these factors, we have adjusted the cut-offs for ALT and bilirubin, and will require that an elevation in AST must be accompanied by an abnormality in another liver function test. Increased cut-off for amylase and lipase in order to detect acute pancreatitis but allow for the presence of mild chronic pancreatitis, which is often seen in methylmalonic acidemia (Hwang 2021).
- Added instructions to stop continuous feeding for 1 hour prior to study blood draws

Version 6.0

- Creation of a single global protocol version; harmonization of the Saudi Arabia specific version of the protocol to include all Version 5 changes above as well as to specify that neurocognitive testing will be performed only in the US due to lack of availability of translations
- Updated inclusion criteria #4 to reflect country specific vaccinations schedules
- Updated inclusion criteria #6 to allow screening hematology to be used to meet criteria
- Updated corticosteroid administration to reflect necessity of administering via g-tube as this patient population may not be able to ingest oral steroids
- Updated sections 5.3.4 and 5.3.5 to clarify which lab tests need to be repeated for rescreening or extended screening windows
- Schedule of events: updated baseline visit labs hematology/chemistry/coagulation/cystatin/eGFR/ammonia to be drawn only if screening labs are drawn > 14 days prior in order to reduce unnecessary duplicate labs; added MMUT antibody testing at several time points to allow additional monitoring
- Added clarification/corrections to endpoint measurements for Propionate Oxidation, Albumin 2A and oral feeding
- Section 7.15: Clarified analytes to be collected for local laboratory analysis

Administrative letter for Protocol V6.0; sent to sites to incorporate prior to Version 7.0 of protocol

- Per the letter dated 03November2021 in regard to the SUSAR of 29Oct2021, the following changes impact LB001-001 Protocol Version 6.0:
  - Patients will not be dosed within 2 weeks of a viral illness.
  - Regular home monitoring with urine dipsticks to enable rapid detection of hematuria or proteinuria between days 7 and 14. [Please note that this was removed in Version 7.0 as patients will be hospitalized during this time]

Version 7.0

- Section 3.2: Added summary of dose selection rational
- Section 4.3: Viral shedding collection extended from 4 weeks to 8 weeks postdosing
- Section 5.1: Incorporation of 4 additional patients in lower-dose cohort 1 (i.e., Group C) for TMA mitigation measures with appropriate adjustments to relevant sections of protocol (eg, statistical methods)
- Section 5.1: Addition of guidance on placement of peripherally inserted central catheter (PICC line) for any patient who does not already have one, to allow for easier blood draws
- Section 5.1: Addition of guidance for prophylactic use of eculizumab to inhibit complement-mediated TMA with concomitant antibiotics for infection prevention
- Section 5.1: Increased length of full-dose prednisolone course from 30 days to 60 days to address persisting abnormal liver function
- Section 5.1: Hospitalization period increased from 4 days to 8 days and daily visits until week 2 to allow for closer monitoring for TMA during the high-risk period for TMA and immediate implementation of treatment, if necessary
- Section 5.1: Additional visits added to allow for additional safety and efficacy sample collection
- Section 5.2: “Any event of TMA” added as trigger for study suspension; AST 2x ULN removed
- Sections 6.2 & 6.3: Clarifications to inclusion criteria #4 (e.g., removal of US and Saudi specific vaccination rules) and exclusion criteria #6, #13, #14, and #15 (e.g., adding allergy to antibiotics and recent viral/bacterial illness as exclusions)
- Section 7.15: Addition of testing for TMA genetic variants
- Section 7.15: Addition of local laboratory tests to monitor for TMA (e.g., blood smear, BMP, LDH/haptoglobin)
- Schedule of events: Revised accordingly to account for additions/changes noted above
- Section 8.1: Addition of TMA experts to the DSMB
- Section 9.4: Clarified that decision to dose escalate will depend on DSMB and Sponsor review of risk/benefit
- Section 11.2.1: TMA added as an AESI to ensure closer monitoring and rapid communication of the event
- Section 12.5: revised language regarding serum methylmalonic acid levels to “average of all predose levels” in the event that additional predosing samples are taken

Version 8.0

- Section 5.1: Based on discussion with the FDA, prophylactic treatment with eculizumab was removed throughout (e.g., synopsis, schedule of events); shifts the management of potential TMA risk from a prevention approach to a monitoring and treatment paradigm
- Section 5.1: Added language to reflect that risk/benefit analysis will be shared with the FDA prior to commencing enrollment in cohort 2
- Section 5.1: Study Design updated to clarify the monitoring regimen for TMA, and the review of laboratory results during the high-risk period
- Section 5.2: Updated study suspension rules to remove requirement for Grade 4 or higher AEs to be assessed as related to hLB-001 by investigator/sponsor (reflects a more conservative standard to evaluate adverse events that may occur)
- Section 5.3: Patient Staggering added to clarify/summarize the patient staggering in each cohort
- Section 6.2: Amended inclusion criteria #4 to extend the window for vaccination from 2 weeks to more than 6 weeks before or after hLB-001 dosing
- Section 6.3: Added history of deep vein thrombosis or coagulopathy as exclusion criteria #16
- Section 7.15: Laboratory tests amended to clarify C3 and C4 can be analyzed locally and clarify prioritization of blood sample collection in cases of insufficient blood volume
- Schedule of events: Added prophylactic meningococcal vaccination to screening period
- Schedule of events: Added additional time points for monitoring of C3 and C4 (related to TMA)
- Section 10.4: Amended to clarify when eculizumab is to be administered and at what dose schedule in the event of TMA
- Section 11.2.1: Definition of TMA removed to remove any doubt regarding when treatment for TMA should start, should it be experienced; clarification of review of laboratory results, signs and symptoms of TMA, and treatment approach to TMA

Version 8.1

- Section 4.3 and schedule of events: Increased length of viral shedding studies to 52 weeks
- Section 10.2: Amended language regarding normal levels of cortisol

## hLB-001 Sequence

ttggccactccctctctgcgcgctcgctcgctcactgaggccgcccgggcaaagcccgggcgtcgggcgacctttggtcgcccggcctcagtgagcgagcgagcgcgcagagagggagtggccaactccatcactaggggttccttacgtaATGttaTGctccatgaaagtggattttattatcctcatcatgcagatgagaatattgagacttatagcggtatgcctgagccccaaagtactcagagttgcctggctccaagatttataatcttaaatgatgggactaccatccttactctctccatttttctatacgtgagtaatgttttttctgttttttttttttctttttccattcaaactcagtgcacttgttgagcttgtgaaacacaagcccaaggcaacaaaagagcaactgaaagctgttatggatgatttcgcagcttttgtagagaagtgctgcaaggctgacgataaggagacctgctttgccgaggaggtactacagttctcttcattttaatatgtccagtattcatttttgcatgtttggttaggctagggcttagggatttatatatcaaaggaggctttgtacatgtgggacagggatcttattttacaaacaattgtcttacaaaatgaataaaacagcactttgtttttatctcctgctctatGgtgccatactgttaaatgtttataatgcctgttctgtttccaaatttgtgatgcttatgaatattaataggaatatttgtaaggcctgaaatattttgatcatgaaatcaaaacattaatttatttaaacatttacttgaaatgtggtggtttgtgatttagttgattttataggctagtgggagaatttacattcaaatgtctaaatcacttaaaattgccctttatggcctgacagtaacttttttttattcatttggggacaactatgtccgtgagcttccgtccagagattatagtagtaaattgtaattaaaggatatgatgcacgtgaaatcactttgcaatcatcaatagcttcataaatgttaattttgtatcctaatagtaatgctaatattttcctaacatctgtcatgtctttgtgttcagggtaaaaaacttgttgctgcaagtcaagctgccttaggcttaggcagcggcgccaccaacttcagcctgctgaaacaggccggcgacgtggaagagaaccctggccccctgagagccaaaaaccagctgttcctgctgagcccccactatctgagacaggtcaaagaaagttccgggagtagactgatccagcagagactgctgcaccagcagcagccactgcatcctgagtgggccgctctggccaagaaacagctgaagggcaaaaacccagaagacctgatctggcacactccagaggggatttcaatcaagcccctgtacagcaaaagggacactatggatctgccagaggaactgccaggagtgaagcctttcacccgcggaccttacccaactatgtatacctttcgaccctggacaattcggcagtacgccggcttcagtactgtggaggaatcaaacaagttttataaggacaacatcaaggctggacagcagggcctgagtgtggcattcgatctggccacacatcgcggctatgactcagataatcccagagtcaggggggacgtgggaatggcaggagtcgctatcgacacagtggaagatactaagattctgttcgatggaatccctctggagaaaatgtctgtgagtatgacaatgaacggcgctgtcattcccgtgctggcaaacttcatcgtcactggcgaggaacagggggtgcctaaggaaaaactgaccggcacaattcagaacgacatcctgaaggagttcatggtgcggaatacttacatttttccccctgaaccatccatgaaaatcattgccgatatcttcgagtacaccgctaagcacatgcccaagttcaactcaattagcatctccgggtatcatatgcaggaagcaggagccgacgctattctggagctggcttacaccctggcagatggcctggaatattctcgaaccggactgcaggcaggcctgacaatcgacgagttcgctcctagactgagtttcttttggggaattggcatgaacttttacatggagatcgccaagatgagggctggccggagactgtgggcacacctgatcgagaagatgttccagcctaagaactctaagagtctgctgctgcgggcccattgccagacatccggctggtctctgactgaacaggacccatataacaatattgtcagaaccgcaatcgaggcaatggcagccgtgttcggaggaacccagagcctgcacacaaactcctttgatgaggccctggggctgcctaccgtgaagtctgctaggattgcacgcaatacacagatcattatccaggaggaatccggaatcccaaaggtggccgatccctggggaggctcttacatgatggagtgcctgacaaacgacgtgtatgatgctgcactgaagctgattaatgaaatcgaggaaatggggggaatggcaaaggccgtggctgagggcattccaaaactgaggatcgaggaatgtgcagctaggcgccaggcacgaattgactcaggaagcgaagtgatcgtcggggtgaataagtaccagctggagaaagaagacgcagtcgaagtgctggccatcgataacacaagcgtgcgcaatcgacagattgagaagctgaagaaaatcaaaagctcccgcgatcaggcactggccgaacgatgcctggcagccctgactgagtgtgctgcaagcggggacggaaacattctggctctggcagtcgatgcctcccgggctagatgcactgtgggggaaatcaccgacgccctgaagaaagtcttcggagagcacaaggccaatgatcggatggtgagcggcgcttatagacaggagttcggggaatctaaagagattaccagtgccatcaagagggtgcacaagttcatggagagagaagggcgacggcccaggctgctggtggcaaagatgggacaggacggacatgatcgcggagcaaaagtcattgccaccgggttcgctgacctgggatttgacgtggatatcggccctctgttccagacaccacgagaggtcgcacagcaggcagtcgacgctgatgtgcacgcagtcggagtgtccactctggcagctggccataagaccctggtgcctgaactgatcaaagagctgaactctctgggcagaccagacatcctggtcatgtgcggcggcgtgatcccaccccaggattacgaattcctgtttgaggtcggggtgagcaacgtgttcggaccaggaaccaggatccctaaggccgcagtgcaggtcctggatgatattgaaaagtgtctggaaaagaaacagcagtcagtgtaacatcacatttaaaagcatctcaggtaactatattttgaattttttaaaaaagtaactataatagttattattaaaatagcaaagattgaccatttccaagagccatatagaccagcaccgaccactattctaaactatttatgtatgtaaatattagcttttaaaattctcaaaatagttgctgagttgggaaccactattatttctattttgtagatgagaaaatgaagataaacatcaaagcatagattaagtaattttccaaagggtcaaaattcaaaattgaaaccaaagtttcagtgttgcccattgtcctgttctgacttatatgatgcggtacacagagccatccaagtaagtgatggctcagcagtggaatactctgggaattaggctgaaccacatgaaagagtgctttatagggcaaaaacagttgaatatcagtgatttcacatggttcaacctaatagttcaactcatcctttccattggagaatatgatggatctaccttctgtgaactttatagtgaagaatctgctattacatttccaatttgtcaacatgctgagctttaataggacttatcttcttatgacaacatttattggtgtgtccccttgcctagcccaacagaagaattcagcagccgtaagtctaggacaggcttaaattgttttcactggtgtaaattgcagaaagatgatctaagtaatttggcatttattttaataggtttgaaaaacacatgccattttacaaataagacttatatttgtccttttgtttttcagcctaccatgagaataagagaaagaaaatgaagatcaaaagcttattcatctgtttttctttttcgttggtgtaaagccaacaccctgtctaaaaaacataaatttctttaatcattttgcctcttttctctgtgcttcaattaataaaaaatggaaagaatctaatagagtggtacagcactgttatttttcaaagatgtgttgtacgtaaggaacccctagtgatggagttggccactccctctctgcgcgctcgctcgctcactgaggccgggcgaccaaaggtcgcccgacgcccgggctttgcccgggcggcctcagtgagcgagcgagcgcgcagagagggagtggccaa

## Additional exploratory endpoints

- Hospitalizations for MMA-related complications (number, reason for, and duration of MMA related hospitalizations from dosing through week 52 end-of-study visit with comparison to 1year period predosing)
- Surgical liver and/or kidney transplant (number of patients with liver, kidney, or combined liver-kidney transplants)
- Growth parameters (change in age-specific *z*-scores for weight, height/length, and head circumference from predosing to week 52 end-of-study visit as determined from standard growth curves)
- Vineland Adaptation Behavior Score (change in patient score from predosing to week 52 end-of-study visit)
- Parenting Stress Index (change in parenting total stress score (PSI-IV, 36-item short form) from predosing to week 52 end-of-study visit)
- Healthcare utilization (number of Emergency Room visits and unscheduled physician visits from dosing through week 52 end-of-study visit with comparison to 1-year period predosing)
- Neurodevelopmental status (change in age-appropriate testing score from predosing to week 52 end-of-study visit, eg, Bayley, Wechsler) [note that these will be collected in the US only]
- Protein intake (determined from 3-day nutrition diary at week 52 end-of-study visit)
- Oral feeding (determined from 3-day nutrition diary at week 52 end-of-study visit)
- MMA-related medication use (prescription medications started/discontinued or change in dose other than weight-related adjustments)
- Exploratory serum/plasma pharmacodynamic biomarkers associated with MMA

## Supplementary Table 1. Genetic testing results

| **Participant** | **Testing panel/ gene(s) tested post treatment** | **Results** |
| --- | --- | --- |
| Participant 1 | None | - N/A |
| Participant 2 | TMA Complete Genetic Panel^a^ | - Heterozygous, synonymous variant (c.1077T>C, p.Ile359Ile) of unknown significance in exon 2 of *C3AR1* - Heterozygous, missense variant (c.1558G>A, p.Gly530Ser) in exon 13 of *C2* (likely benign) - Heterozygous for five polymorphisms in *MCP/CD46*:  (i) c.-652A>G, (ii) c.-366A>G, (iii) c.989-78G>A,  (iv) c.1127+638G>A, and (v) c.*783T>C |
| Participant 3 | TMA Complete Genetic Panel^a^ | - Heterozygous missense polymorphism (c.3097G>A, p.Ala1033Thr) in exon 24 of *ADAMTS13 (*benign/likely benign) - Heterozygous synonymous variant (c.1674G>A, p.Ser558Ser) of unknown significance in exon 12 of *DGKE* - Heterozygous missense variant (c.1217G>A, p.Arg406His) in exon 11 of *CFI* (likely benign) - Three heterozygous polymorphisms in *CFH*: (i) variant in the promoter (chr1:196620917, C>T), (ii) silent variant in exon 13 (c.2016A>G, p.Gln672Gln), and (iii) missense variant in exon 18 (c.2808G>T, p.Glu936Asp) - Heterozygous for c.-652A>G in *MCP/CD46* |
| Participant 4 | Factor V Leiden variant; prothrombin II gene | - Tested variants not found   - F5 c.1601G>A p.Arg534Gln, not detected   - Prothrombin II G20210A variant (g.21538G>A [c.*97G>A]), not detected |
|  | aHUS Genetic Panel^b^ | - Heterozygous missense variant (c.1058C>T, p.Ala353Val) in exon 8 of *MCP/CD46*^c^ |

^a^Machaon Diagnostics. TMA Complete Genetic Panel. <https://www.machaondiagnostics.com/test/tma-complete-genetic-panel/>

^b^Machaon Diagnostics. aHUS Genetic Panel. <https://www.machaondiagnostics.com/test/ahus-genetic-panel/>

^c^“Historically, [Machaon Diagnostics] reported [the *MCP/CD46* c.1058C>T, p.Ala353Val] variant as pathogenic. However, although it is rare according to the 1000 Genomes database (minor allele frequency = 0.004), it is not rare according to the gnomAD database (minor allele frequency = 0.01541).” Participant 4 is of Finnish ancestry (maternal side) and this variant “can be as common as 0.06 in the Finnish population.” “Fang et al. [2008] generated cell lines expressing the p.Ala353Val mutant MCP/CD46 protein and showed that these cells were defective in regulating complement.” – quotations from the aHUS Genetic Panel test report for Participant 4.

*ADAMS13,* ADAM metallopeptidase with thrombospondin type 1 motif 13*;* aHUS, atypical hemolytic uremic syndrome; *C2*, complement component 2; *C3AR1*, complement component 3a receptor 1; *CFH*, complement factor H; *CFI*, complement factor I; *DGKE*, diacylglycerol kinase epsilon; *F5*, coagulation factor V; *MCP/CD46*, membrane cofactor protein/cluster of differentiation 46; N/A, not applicable; TMA, thrombotic microangiopathy.


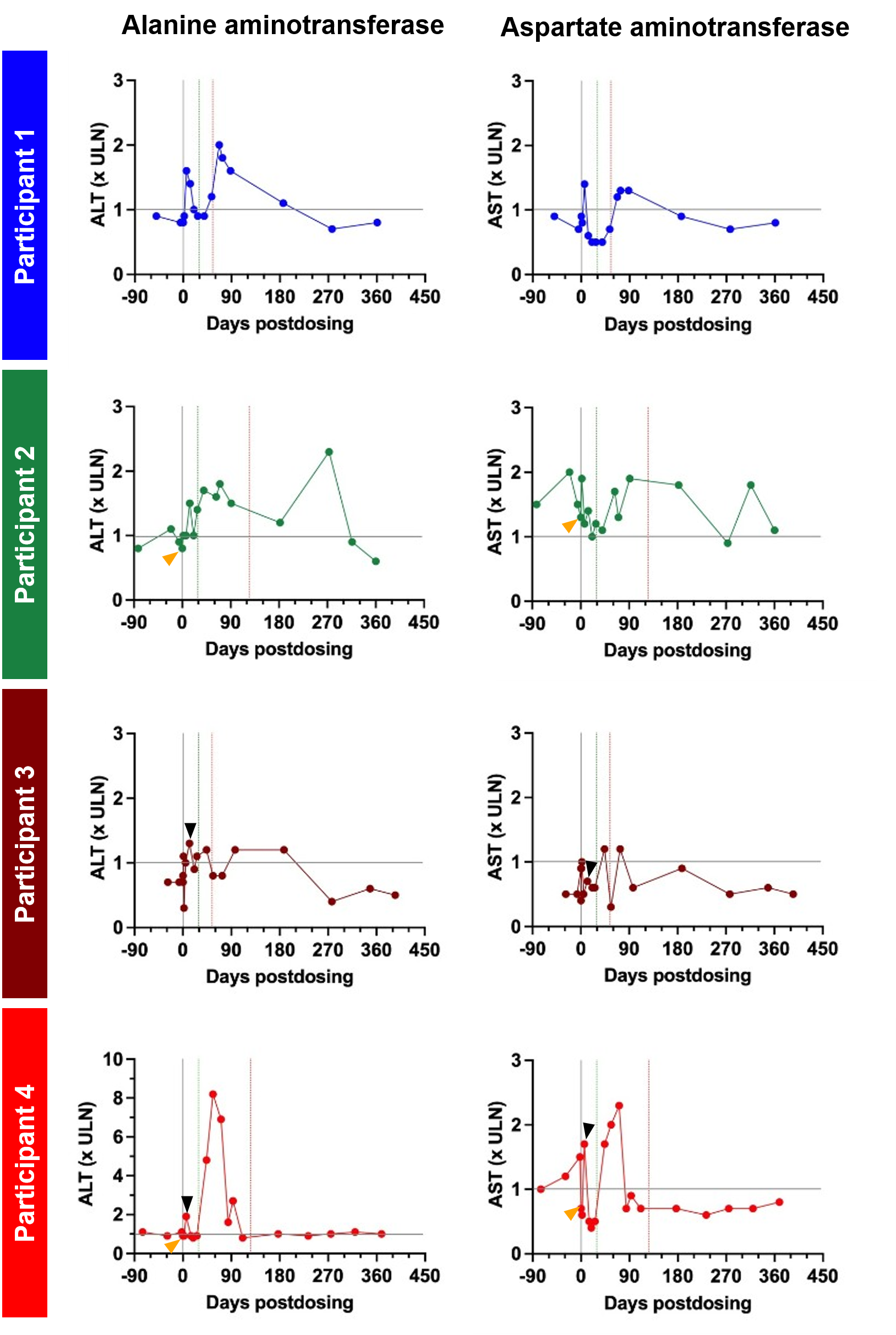


## Supplementary Fig. 1. Levels of serum alanine aminotransferase (ALT) and aspartate aminotransferase (AST) in individual participants.

For Participant 2, cytokine release syndrome occurred from 1 to 2 days postinfusion (orange arrowhead); ALT was below the ULN but AST was above 1x ULN with subsequent elevations greater than 1x ULN in both after the event had resolved. For Participant 3, TMA was documented 12 days after hLB-001 infusion (black arrowhead) and persisted until day 18 postinfusion. During this period, ALT was elevated to greater than 1x ULN, while AST remained below 1x ULN). For Participant 4, cytokine release syndrome occurred during days 1 to 4 after infusion (orange arrowhead); both ALT and AST were below the ULN on days 1 and 3 (not reported on days 2 and 4). TMA was documented from day 6 to day 27 postinfusion; although ALT and AST transiently increased above 1x ULN on day 7 (black arrowhead), values were below 1× ULN during the rest of this period. Peak ALT (about 8x ULN) and AST (slightly above 2x ULN) occurred around day 57 postinfusion. Green dotted vertical line indicates start of steroid taper. Pink dotted vertical line indicates last steroid dose. Note: The y-axis ALT (x ULN) range for Participant 4, who required the longest extended steroid course for resolution of significantly increased transaminases, is different than the other graphs.

ALT, alanine aminotransferase; AST, aspartate aminotransferase; TMA, thrombotic microangiopathy; ULN, upper limit of normal.


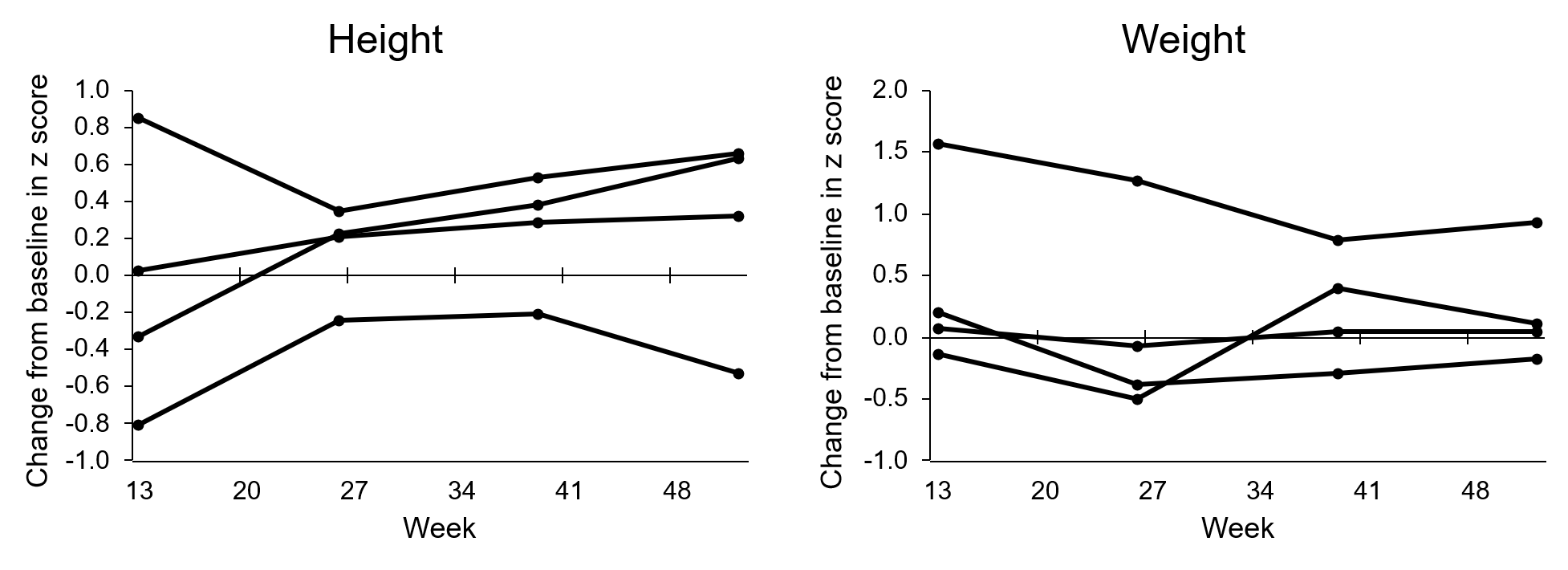


## Supplementary Fig. 2. Change from baseline in height and weight.

Due to the rarity of gene editing studies involving pediatric participants with MMA, and to protect participant identity and maintain confidentiality, changes in height and weight over the course of the trial are presented without participant labels.

MMA, methylmalonic acidemia.

**
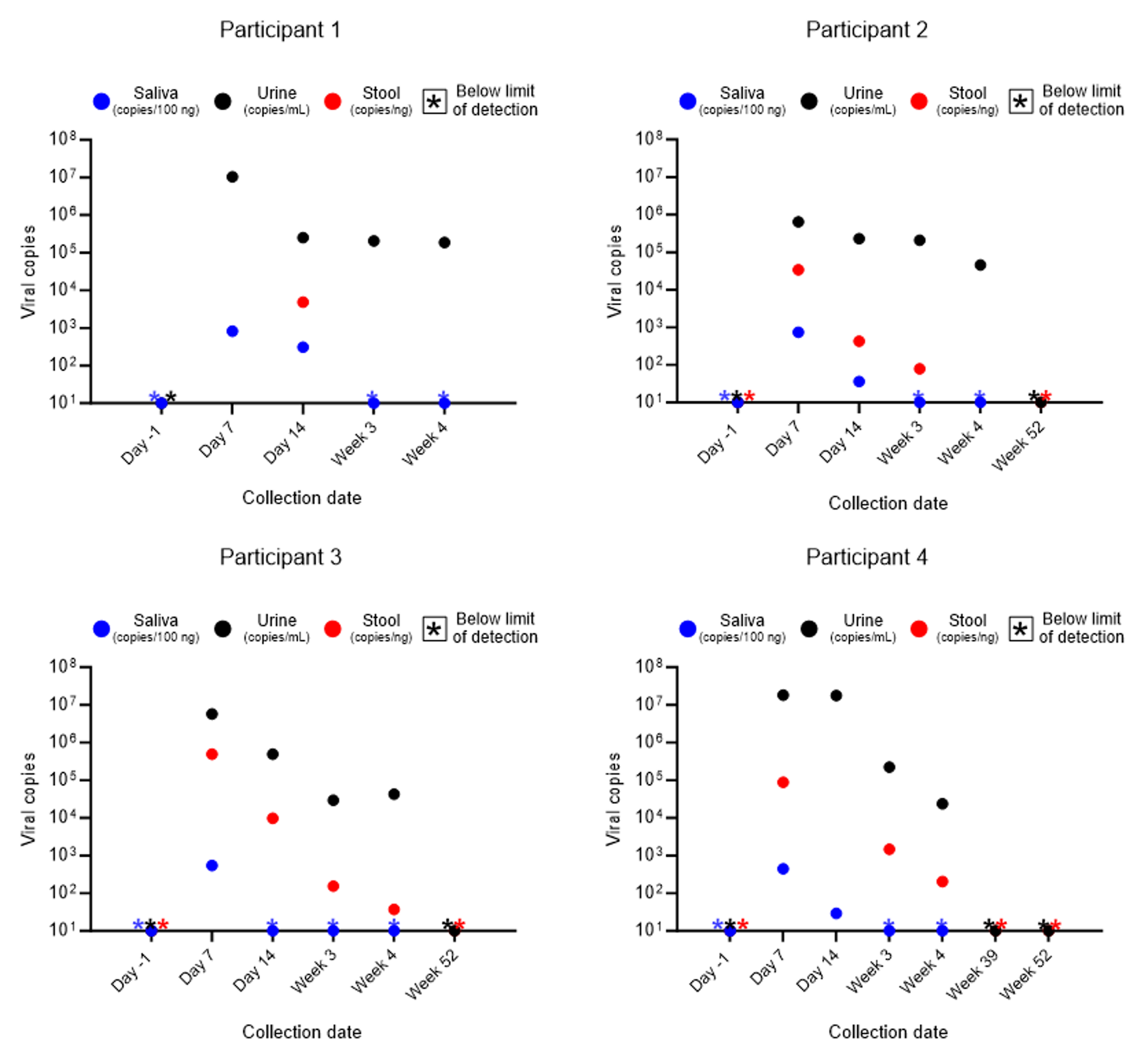
**

## Supplementary Fig. 3. Viral shedding in individual participants.

Lower limit of quantification is 25 copies of double-stranded DNA. Not all samples were available at all time points.
